# Timing and sequence of primary tooth eruption in children with cleft lip and palate

**DOI:** 10.1590/S1678-77572010000300004

**Published:** 2010

**Authors:** Tatiana Yuriko KOBAYASHI, Márcia Ribeiro GOMIDE, Cleide Felício de Carvalho CARRARA

**Affiliations:** 1 DDS, MSc, PhD student, Department of Pediatric Dentistry, Orthodontics and Public Health, Bauru School of Dentistry, University of São Paulo, Bauru, SP, Brazil.; 2 DDS, MSc, PhD, Pediatric Dentistry Sector, Hospital for Rehabilitation of Craniofacial Anomalies, University of São Paulo, Bauru, SP, Brazil.; 3 DDS, MSc, PhD student, Pediatric Dentistry Sector, Hospital for Rehabilitation of Craniofacial Anomalies, University of São Paulo, Bauru, SP, Brazil.

**Keywords:** Tooth eruption, Primary dentition, Cleft lip, Cleft palate

## Abstract

**Objective:**

To determine the timing and sequence of eruption of primary teeth in children with complete bilateral cleft lip and palate.

**Material and Methods:**

This cross-sectional study was conducted at the Hospital for Rehabilitation of Craniofacial Anomalies of the University of São Paulo, Bauru, SP, Brazil, with a sample of 395 children (128 girls and 267 boys) aged 0 to 48 months, with complete bilateral cleft lip and palate

**Results:**

Children with complete bilateral clefts presented a higher mean age of eruption of all primary teeth for both arches and both genders, compared to children without clefts. This difference was statistically significant for all teeth, except for the maxillary first molar. Mean age of eruption of most teeth was lower for girls compared to boys. The greatest delay was found for the maxillary lateral incisor, which was the eighth tooth of children with clefts of both genders. Analyzing by gender, the maxillary lateral incisor was the eighth tooth to erupt in girls and the last in boys.

**Conclusion:**

The results suggest an interference of the cleft on the timing and sequence of eruption of primary teeth.

## INTRODUCTION

The period of eruption of primary teeth is influenced by several factors, such as length of gestational period^25^ , disease^7^ , gender^3,14^ , race^14,15^ , nutrition and general growth^10^. In addition, cleft lip and palate may also influence the timing and sequence of eruption of both primary^4,11,12,18^ and permanent teeth^2,13,21^. This malformation causes injury to the face in many different manners, depending on its occurrence, resulting in morphological and functional incapacity to variable extents. However, as the alveolar ridge is not always affected in all cleft types, it might be assumed that the different types of clefts may not exert the same influence on tooth eruption in these patients, as observed by some authors^5,22,23^.

Only few studies have mentioned the timing of eruption of primary teeth in children with clefts. Fishaman^5^ (1970) evaluated both primary and permanent dentitions and observed an evident delay in the timing of tooth eruption in patients with different types of clefts, the greatest delay occurring for bilateral clefts and for the maxillary lateral incisor on the cleft side.

Analyzing the influence of the cleft on the eruption of maxillary incisors, Kramer, et al.^12^ (1989) observed a significant delay in the eruption of the lateral incisor on the cleft side. The delay increased when a palatal cleft was also present. A delay of approximately 8 months was observed in the eruption of primary maxillary lateral incisors when positioned at the distal segment in children with cleft lip and alveolus and 13 months in children with cleft lip and palate; when the lateral incisor is positioned at the premaxilla, the delay was approximately 4 months longer. Kramer, et al.^12^ (1993) studied the eruption of primary canines and molars, and observed that the only significant delay affected the first molars on the cleft side, both in the maxilla and mandible, in complete unilateral cleft lip and palate^11^.

Pöyry and Ranta^19^ (1985) evaluated the eruption of primary teeth in different types of clefts, comparing the results with the findings of children without clefts. In patients with clefts, all teeth on Timing and sequence of primary tooth eruption in children with cleft lip and palate the cleft area erupted later when compared to their homologues on the noncleft side.

In order to contribute to the studies on tooth eruption, this study investigated the chronology and sequence of eruption of primary teeth in white Brazilian children with complete bilateral cleft lip and palate. The expected results for this study would be identifying differences between children with children complete bilateral cleft lip and palate, and children without cleft, on the issues studied.

## MATERIAL AND METHODS

The study sample of this cross-sectional study was composed of 395 white Brazilian children (128 girls and 267 boys) aged 0 to 48 months with complete bilateral cleft lip and palate, attending the Hospital for Rehabilitation of Craniofacial Anomalies of the University of São Paulo (HRAC/USP), Bauru, SP, Brazil. The duration of the surgical protocol for correction of the lip is about 3 months and the palate is more than 12 months.

Syndromic children were not included in this study because of possible influence on tooth eruption inherent to certain syndromes. The research project was approved by the HRAC/ USP’s institutional review board. Individuals were considered white if they presented skin color ranging from white to dark and straight to lightly curly or curly hair type^1^.

A previously trained single observer performed the examination under natural light. The teeth were considered as erupted whenever there was any portion of the crown showing above the gingival barrier. Teeth that were not present in the oral cavity were considered as unerupted unless the child’s caretaker provided information on tooth extraction. Natal and neonatal teeth were not considered in this study.

After data collection, children were assigned to 49 age and gender groups. The mean age of eruption for each tooth was calculated using the Kärber method, as modified by Hayes and Mantel^9^ (1958). The results obtained for children with bilateral clefts were compared to the results of Vono, et al.^26^ (1972). Children without clefts were taken as the control group.

Statistical analysis was performed using the Student’s “t” test at a significance level of 0.05 (p<0.05) to assess possible differences in the age of eruption of primary teeth between genders and between the cleft and control groups, for both the maxilla and mandible.

## RESULTS

From the 395 patients examined in this study, 32.4% were girls and 67.6% were boys. The results obtained for the mean ages of eruption of primary teeth are presented in Table 1. Tables 2-4 present the results of comparisons of mean ages of eruption between genders and between children with and Timing and sequence of primary tooth eruption in children with cleft lip and palate without clefts. The sequence of eruption obtained for each gender is shown in Figure 1.

**Table 1:** Mean, Standard Deviation (SD), and Standard Error (SE), in months, of eruption of maxillary and mandibular primary teeth in girls and boys

**TOOTH**	**GIRLS**			**BOYS**		
	**MEAN**	** SD**	**SE**	**MEAN**	** SD**	**SE**
						
Maxillary central incisor	12.5	5.62	0.53	14.53	9.64	0.67
Maxillary lateral incisor	25.58	12.42	0.9	31.16	13.16	1.26
Maxillary canine	22.2	5.14	0.65	21.33	4.04	0.4
Maxillary first molar	15.42	3.16	0.49	17.03	3.44	0.31
Maxillary second molar	30.5	4	0.29	29.42	3.6	0.29
Mandibular central incisor	10.27	2.05	0.25	9.89	3.94	0.38
Mandibular lateral incisor	15.52	4.19	0.5	16.20	4.67	0.37
Mandibular canine	21.45	4.11	0.6	21.94	4.08	0.40
Mandibular first molar	16.2	2.57	0.29	18.28	3.15	0.32
Mandibular second molar	28.75	1.98	0.25	28.44	2.87	0.22

**Table 2:** Comparison of mean ages of eruption of primary teeth for girls versus boys. Statistically significant difference at p<0.05

**TOOTH**	**GIRLS**	**BOYS**	**p Value**
			
Maxillary central incisor	12.5	14.53	0.0181
Maxillary lateral incisor	25.58	31.16	0.0004
Maxillary canine	22.2	21.33	0.25^ns^
Maxillary first molar	15.42	17.03	0.0061
Maxillary second molar	30.5	29.42	0.0088
Mandibular central incisor	10.27	9.89	0.40^ns^
Mandibular lateral incisor	15.52	16.20	0.27^ns^
Mandibular canine	21.45	21.94	0.50^ns^
Mandibular first molar	16.2	18.28	0.0000
Mandibular second molar	28.75	28.44	0.3529^ns^

ns = non significant

**Figure 1:** Sequence of eruption of primary teeth in children with complete bilateral cleft lip and palate

**SEQUENCE**	**GIRLS**	**BOYS**
1st	Mandibular central incisor	Mandibular central incisor
2nd	Maxillary central incisor	Maxillary central incisor
3rd	Maxillary first molar	Mandibular lateral incisor
4th	Mandibular lateral incisor	Maxillary first molar
5th	Mandibular first molar	Mandibular first molar
6th	Mandibular canine	Maxillary canine
7th	Maxillary canine	Mandibular canine
8th	Maxillary lateral incisor	Mandibular second molar
9th	Mandibular second molar	Maxillary second molar
10th	Maxillary second molar	Maxillary lateral incisor

## DISCUSSION

The timing and sequence of eruption of primary teeth may differ somewhat among populations. Studies have also shown a delay in eruption in patients with malformations and malocclusion^2,4,6,14,17,24^.

A delay in eruption of primary teeth has been demonstrated in previous studies with children with cleft lip and palate^2,4,5,6,12^.

There are several studies on tooth eruption and formation in patients with clefts, in which the authors did not separate the samples according to cleft type^5,8,20^ , which impairs comparisons of the present results. Therefore, only patients with complete bilateral cleft lip and palate were evaluated in this study, so that the data obtained for the mean ages of tooth eruption were compared just between the genders and between the cleft and control groups. Considering that the presence of cleft and child’s gender might influence the timing of tooth eruption, data were separated between genders.

Girls showed a lower mean age of eruption for most maxillary and mandibular teeth compared to boys, with significant differences for the maxillary central incisors, lateral incisors and first molars and mandibular first molars. The maxillary second molar erupted earlier in boys, which was significantly different (Table 2).

Gender differences in the timing of tooth eruption in children with bilateral clefts (i.e. earlier eruption in girls) were in agreement with previous studies of cleft populations, confirming the need of quantitative rules for each gender^2,4^. However, some teeth showed earlier eruption in boys, which can be justified by the lower number of girls evaluated in relation to the number of boys, due to the higher frequency of complete bilateral cleft lip and palate among boys^2,4^.

Comparison of data between the study and control groups revealed that, in girls, eruption of all teeth was significantly delayed in the group with cleft, except for the maxillary and mandibular first molars (Table 3). On the other hand, in boys, eruption of all teeth was significantly delayed in the cleft group (Table 4).

**Table 3:** Comparison of mean ages of eruption of primary teeth of girls with complete bilateral cleft lip and palate (X-2005) versus girls without clefts (X-Vono ^26^ , 1972). Statistically significant difference at p<0.05

**TOOTH**	**X - (2005)**	**X - Vono ^26^ (1972)**	**p VALUE**
			
Maxillary central incisor	12.5	10.37	0.0002
Maxillary lateral incisor	25.58	12.17	0.0000
Maxillary canine	22.2	18.85	0.0000
Maxillary first molar	15.42	15.19	0.6643^ns^
Maxillary second molar	30.5	26.41	0.0000
Mandibular central incisor	10.27	8.37	0.0000
Mandibular lateral incisor	15.52	14.03	0.0072
Mandibular canine	21.45	19.48	0.0033
Mandibular first molar	16.2	15.85	0.3452^ns^
Mandibular second molar	28.75	25.11	0.0000

ns = non significant

**Table 4:** Comparison of mean ages of eruption of primary teeth of boys with complete bilateral cleft lip and palate (X-2005) versus boys without clefts (X-Vono ^26^ , 1972). Statistically significant difference at p<0.05

**TOOTH**	**X - (2005)**	**X - Vono ^26^ (1972)**	**p VALUE**
			
Maxillary central incisor	14.53	9.47	0.0000
Maxillary lateral incisor	31.16	11.21	0.0000
Maxillary canine	21.33	18.18	0.0000
Maxillary first molar	17.03	15.62	0.0001
Maxillary second molar	29.42	26.72	0.0000
Mandibular central incisor	9.89	8.00	0.0000
Mandibular lateral incisor	16.20	13.00	0.0000
Mandibular canine	21.94	19.13	0.0000
Mandibular first molar	18.28	16.07	0.0000
Mandibular second molar	28.44	25.67	0.0000

The most significant delay in the cleft group compared to the control group was observed for the maxillary lateral incisor, which erupted 13.41 and 19.95 months later in girls and boys, respectively.

The delay in the mean age of eruption of the maxillary lateral incisor may have a multifactorial etiology and can also be related to the intrinsic and extrinsic factors that contribute with tooth development of individuals to clefts. Local factors include scar tissue after surgical repair, delayed formation of crown and root, poor blood circulation after surgery^19^ , lack of space in the maxilla^17^ and lack of bone support^16^.

The sequence of eruption of primary teeth in the study group varied between genders, as shown in Figure 1. The last tooth to erupt in girls was the maxillary second molar and the maxillary lateral incisor in boys. The sequence of tooth eruption in the mandible did not differ from the control group.

In the maxillary arch of girls, the canine erupted before the lateral incisor, whereas in the maxillary arch of the control group and boys, the canine and second molar erupted before the lateral incisor.

## CONCLUSION

The findings of the present study suggest that the cleft affects the timing and sequence of eruption of primary teeth, which corroborates the findings in the literature evaluating the chronological sequence of eruption on reliable samples of patients with clefts^2,4^. Knowledge of the mean timing and sequence of eruption of primary teeth in children with complete bilateral cleft lip and palate adds importance information to professionals working in the rehabilitation process of patients with malformations, in order to provide parents with information about inherent characteristics of these patients.
